# Endogenous amdoparvovirus-related elements reveal insights into the biology and evolution of vertebrate parvoviruses

**DOI:** 10.1093/ve/vey026

**Published:** 2018-11-12

**Authors:** Judit J Pénzes, Soledad Marsile-Medun, Mavis Agbandje-McKenna, Robert James Gifford

**Affiliations:** 1 University of Florida McKnight Brain Institute, 1149 Newell Dr, Gainesville, USA; 2 Agrocampus Ouest, 65 Rue de Saint-Brieuc, Rennes, France; 3 MRC-University of Glasgow Centre for Virus Research, 464 Bearsden Road, Glasgow, UK

**Keywords:** parvovirus, amdoparvovirus, paleovirology, protoparvovirus, endogenous virus

## Abstract

Amdoparvoviruses (family *Parvoviridae:* genus *Amdoparvovirus*) infect carnivores, and are a major cause of morbidity and mortality in farmed animals. In this study, we systematically screened animal genomes to identify endogenous parvoviral elements (EPVs) disclosing a high degree of similarity to amdoparvoviruses, and investigated their genomic, phylogenetic and protein structural features. We report the first examples of full-length, amdoparvovirus-derived EPVs in the genome of the Transcaucasian mole vole (*Ellobius lutescens*). We also identify four EPVs in mammal and reptile genomes that are intermediate between amdoparvoviruses and their sister genus (*Protoparvovirus*) in terms of their phylogenetic placement and genomic features. In particular, we identify a genome-length EPV in the genome of a pit viper (*Protobothrops mucrosquamatus*) that is more similar to a protoparvovirus than an amdoparvovirus in terms of its phylogenetic placement and the structural features of its capsid protein (as revealed by homology modeling), yet exhibits characteristically amdoparvovirus-like genome features including: (1) a putative middle ORF gene; (2) a capsid gene that lacks a phospholipase A2 domain; (3) a genome structure consistent with an amdoparvovirus-like mechanism of capsid gene expression. Our findings indicate that amdoparvovirus host range extends to rodents, and that parvovirus lineages possessing a mixture of proto- and amdoparvovirus-like characteristics have circulated in the past. In addition, we show that EPV sequences in the mole vole and pit viper encode intact, expressible replicase genes that have potentially been co-opted or exapted in these host species.

## 1. Introduction

Parvoviruses (family *Parvoviridae*) are small, single-stranded DNA viruses that infect vertebrate (subfamily *Parvovirinae*) and invertebrate (subfamily *Densovirinae*) hosts. The small (4–6 kb) genome is encompassed by characteristic terminal palindromic repeats, which form hairpin-like secondary structures characteristic for each genus (Tijssen et al. 2011; Cotmore et al. 2014). Despite exhibiting a low level of sequence similarity, parvovirus genomes are highly conserved in overall structure, containing two large gene cassettes responsible for encoding the nonstructural (NS) and the structural (VP) proteins. The N-terminal region of the minor capsid protein VP1 includes a highly conserved phospholypase A2 (PLA2) motif that is required for escape from the endosomal compartments after entering the host cell (Zadori et al. 2001). The parvovirus capsid is icosahedral with a T = 1 symmetry, displaying a jelly roll fold of conserved β-sheets linked by variable surface loops, designated variable region (VR) I to IX (Chapman and Agbandje-McKenna 2006).

Endogenous parvoviral elements (EPVs) are genomic sequences homologous to parvovriuses that are thought to have been generated when parvoviral DNA sequences were incorporated into the germline of the ancestral host species, such that they were subsequently inherited as host alleles (Holmes 2011; Feschotte and Gilbert 2012). The identification of orthologous EPVs in the genomes of distantly related species demonstrates that these sequences were generated millions of years ago (Belyi et al. 2010; Kapoor et al. 2010; Katzourakis and Gifford 2010; Smith et al. 2016), and they can therefore provide a useful source of retrospective information about the longer-term evolutionary relationships between parvoviruses and their hosts.

*Amdoparvovirus* is a recently defined genus in the family *Parvoviridae* (Cotmore et al. 2014). The type species was originally called Aleutian mink disease virus (AMDV)—hence the genus name. However, AMDV is now considered to represent a variant of the renamed species *Carnivore amdoparvovirus 1* (Cotmore et al. 2014). AMDV causes an immune complex-associated progressive syndrome in American mink (*Neovison vison*) called Aleutian disease or plasmacytosis (Bloom et al. 1994) which is considered to be one of the most important infectious diseases affecting farm-raised mink (Canuti et al. 2016). AMDV infection is known to be widespread in wild mink as well as in farmed animals (Canuti et al. 2015), and related amdoparvoviruses have been identified in other carnivore species, including raccoon dogs, foxes, skunks, and red pandas (Pennick et al. 2007; Li et al. 2011; Shao et al. 2014; LaDouceur et al. 2015; Alex et al. 2018). Findings from metagenomic studies suggest that amdoparvoviruses may infect a broader range of mammalian orders (Lau et al. 2017), but this has yet to be fully demonstrated.

Phylogenetic studies support a common evolutionary origin for amdoparvoviruses and protoparvoviruses (genus *Protoparvovirus*) (Cotmore et al. 2014). Protoparvoviruses infect a wide range of mammalian hosts, encompassing several distinct mammalian orders (Tijssen et al. 2011; Sasaki et al. 2015). For example, rodent protoparvoviruses are known for their oncolytic properties (Marchini et al. 2015), while carnivore and ungulate protoparvoviruses are significant pathogens of domestic pets and livestock (Hueffer and Parrish 2003; Kailasan et al. 2015; Meszaros et al. 2017).

Although they are relatively closely related, amdoparvoviruses and protoparvoviruses are distinguished by certain features of their genomes and replication strategies. In particular, amdoparvovirus mRNAs are transcribed from one single upstream promoter and are polyadenylated at two distinct polyadenylation signals. To provide the VP1 encoding transcript, an intron is spliced out, leaving a short, three amino acid (aa)–encoding exon leader sequence (transcribed from a short, upstream ORF) positioned in-frame with the VP ORF (Qiu et al. 2006). In contrast, the NS- and VP-encoding mRNAs of protoparvoviruses are transcribed from two different promoters, being polyadenylated at a mutual polyadenylation signal close to the 3′ end of the genome. Although splicing has been reported in the protoparvovirus VP-encoding mRNA, this always occurs within the VP ORF itself (Tijssen et al. 2011). Amdoparvoviruses are also unique within the *Parvovirinae* in lacking a PLA2 domain in their VP unique region (VP1u) (Zadori et al. 2001; Cotmore et al. 2014).

In this study, we performed a systematic screen of 688 animal genomes to identify EPVs disclosing similarity to amdoparvoviruses. We identify and characterize six such EPVs, examining their genomic, phylogenetic and protein structural characteristics.

## 2. Methods

### 2.1 *In silico* genome screening

We used the database-integrated genome screening (DIGS) tool (Zhu et al. 2018) to screen whole-genome sequence (WGS) assemblies for EPVs. The DIGS tool provides a framework for similarity-search–based genome screening. It uses the basic local alignment search tool (BLAST) program (Altschul et al. 1997) to systematically screen WGS files for sequences matching to a nucleotide or peptide ‘probe’. Sequences that disclose above-threshold similarity to the probe are extracted and classified, with results being captured in an MySQL relational database (Axmark and Widenius 2015). To identify EPVs, we used parvovirus peptide sequences to screen all 362 vertebrate genome assemblies available in the NCBI WGS database as of the 15 December 2017. Sequences that produced statistically significant matches to these probes were extracted and classified by BLAST-based comparison to a set of reference peptide sequences selected to represent the broad range of diversity in subfamily *Parvovirinae*.

### 2.2 Sequence analyses

Characterization and annotation of EPVs was performed using the Artemis Genome Browser (Carver et al. 2012). Putative peptide sequences encoded by EPVs were inferred and aligned using MUSCLE (Edgar 2004), PAL2NAL (Suyama et al. 2006), and T-coffee Expresso (Armougom et al. 2006). Phylogenies were reconstructed from aa alignments incorporating structural data (at least one high-resolution structure from all available genera), and using maximum likelihood (ML) as implemented in PhyML-3.1, with 1000 bootstrap replicates (Guindon et al. 2010). The RtEV (NS) and the LG (VP) protein substitution models—as selected using ProTest (Abascal et al. 2005)—were used for reconstructing phylogenies.

To detect structural homology, we applied the pGenTHREADER and pDomTHREADER algorithms of the PSIPRED Protein Sequence Analysis Workbench (Lobley et al. 2009). The selected PDB structures were applied as templates for homology modeling, carried out by SWISS-MODEL (Biasini et al. 2014). Polymers of the acquired capsid monomer models were constructed by the Oligomer Generator feature of the Viper web database (http://viperdb.scripps.edu/) (Carrillo-Tripp et al. 2009). The generated polymers were rendered as well as ribbon diagrams compared using PYMOL (Schrödinger).

## 3. Results

### 3.1 Identification and characterization of EPVs

We screened WGS assemblies of 688 animal species for EPVs disclosing a high degree of homology to amdoparvoviruses. Similarity searches using the replicase (NS) and capsid (VP) proteins of AMDV identified six such EPVs (Table 1). These sequences were identified in five distinct vertebrate species, including a reptile—the spotted pit viper—in addition to four mammals. The mammals included three placental species (a rodent and two afrotherians) and one marsupial.

**Table 1.** EPVs characterized in this study.

Species in which identified		Genus/clade	Accession^a^	Element ID^b^	Genes present
Common name	Latin binomial				
Cape hyrax	*Procavia capensis*	AP	ABRQ02031156.1	EPV-AP-ProCap.1	NS-VP
Tasmanian devil	*Sarcophilus harrisii*	AP	AFEY01431940.1	EPV-AP-SarHar.1	NS-VP
Aardvark	*Orycteropus afer*	AP	ALYB01102612.1	EPV-AP-OryAfe.1	NS-VP
Pit viper	*Protobothrops mucrosquamatus*	AP	BCNE02035092.1	EPV-AP-ProMuc.1	NS-M-VP
Mole vole	*Ellobius lutescens*	Amdo	LOEQ01006026.1	EPV-Amdo-EllLut.1	NS-M-VP
Mole vole	*Ellobius lutescens*	Amdo	LOEQ01001077.1	EPV-Amdo-EllLut.2	NS

a Genbank accession numbers indicate genomic scaffolds.

b We applied a systematic approach to naming EPVs. Each element was assigned a unique identifier (ID) constructed the following components: (1) the classifier ‘EPV’; (2) the taxonomic group into which the element is placed; (3) a numeric ID that uniquely identifies the insertion and its orthologous copies within its respective taxonomic group.

Amdo, genus *Amdoparvovirus*; AP, amdo-proto lineage; NS, replicase; VP, capsid; M, middle ORF.

**Figure 1.**
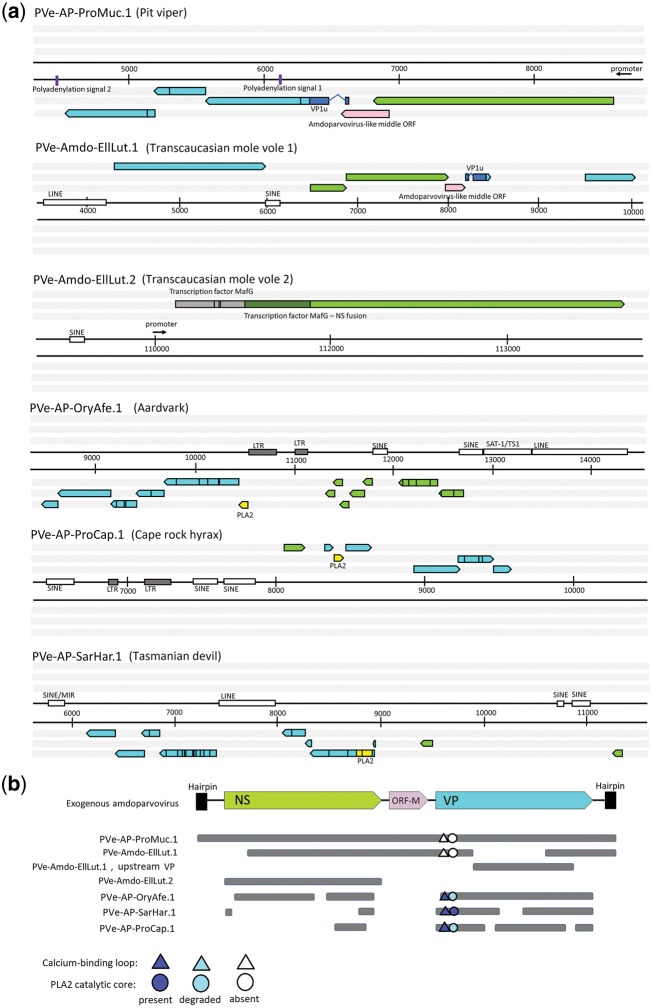
Genomic organization of six amdoparvovirus-like EPVs. (a) Genomic structure of EPV loci, showing features identified in all six frames. Regions of homology to parvovirus proteins are indicated as arrows (NS in green and VP in cyan). Stop codons are indicated by vertical black lines, putative promoters with small black arrows. The characteristic M-ORF homologs of amdoparvoviruses are shown in pink. In the EilLut.2 element, dark green represents the potentially expressed, NS-fused region of the MafG transcription factor. The remaining portion of the MafG pseudogene is shown in grey. (b) Genomic organization of EPVs, shown in relation to a representative amdoparvovirus genome. NS, nonstructural protein; VP, capsid protein; VP1u, VP1 unique region; LINE, long interspersed nuclear element; SINE, short interspersed nuclear element; LTR, long terminal repeat; PLA2, phospholipase A2 domain.

**Figure 2.**
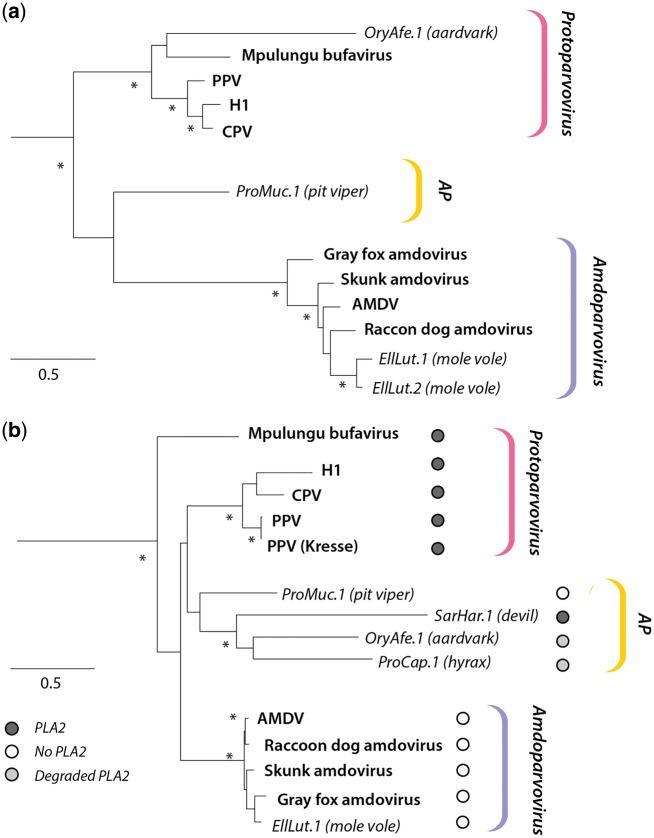
ML phylogenies of amdoparvoviruses, protoparvovirus and EPVs. Phylogenies based on NS (a) and VP (b) peptide sequences. Viral taxa are shown in bold text. The taxa names of EPVs are shown in italics. Brackets to the right indicate viral genera (*Amdoparvovirus*, *Protoparvovirus*) and EPV clades. Asterisks indicate nodes with bootstrap support >90%. The scale bar shows genetic distance in substitutions per site. AMDV, Aleutian mink disease virus; CPV, Canine parvovirus; PPV, Porcine parvovirus; MVM, Minute virus of mice; H1, H-1 parvovirus; AP, Proto-Amdo clade of EPVs. Details of EPVs examined here are contained in Table 1. Supplementary Table S1 contains the accession numbers and other details of amdoparvovirus and protoparvovirus reference sequences.

In all cases, sequences were identified in contigs that were orders of magnitude larger than a parvovirus genome, and it was clear they represented EPVs as opposed to contaminating virus (see later). All six loci were examined using sequence comparison tools to determine their genomic structure relative to reference viruses, and to identify the locations of other genomic features, such as promoters, polyadenylation signals, and transposable element insertions (Fig. 1). Comparisons of genomic sequences flanking EPVs established that all are present at distinct locations, and were generated in distinct germline incorporation events. To infer the evolutionary relationships of these elements to contemporary parvoviruses, we reconstructed ML phylogenies using conserved regions of their putative NS and VP peptide sequences (Fig. 2).

### 3.2 Amdoparvovirus-derived EPVs in a rodent genome

We identified two EPVs derived from amdoparvoviruses in the genome of the Transcaucasian mole vole (*Ellobius lutescens*) (Table 1). ML phylogenes reconstructed using the putative peptide sequences encoded by these elements showed that both are closely related to one another, and group robustly within the clade defined by exogenous amdoparvoviruses (Fig. 2). The first (EPV-Amdo-EllLut.1) spanned a near complete genome containing both the NS and VP genes, while the second (EPV-Amdo-EllLut.2) spanned the majority of the NS gene, with no identifiable VP present (see Fig. 1). These elements are hereafter referred to as EllLut.1 and EllLut.2, respectively.

EllLut.1 is integrated into a locus that is homologous to mouse chromosome 12. This element is derived from genome-length nucleic acid. It contains a putative 3′ untranslated region (UTR) that exhibits homology to the 3′UTR of AMDV, and contains inverted repeats capable of folding into a stem loop structure (Supplementary Fig. S1).

The putative NS ORF of EllLut.1 has gaps relative to AMDV: the N-terminal region of NS is absent up to residue 28 G. The NS ORF is flanked on either side by regions of VP homology (see Fig. 1). A partial VP ORF could be identified downstream of the NS gene, corresponding to the VP1u and the VP2 N-terminal, as well as nucleotides encoding the last 173 aa of the C-terminus. However, a large part of the ORF is missing due to an assembly gap. A region of VP homology—spanning residues 59–600—could be identified upstream of the NS ORF, encompassed by LINE and SINE elements. Only 60 aa of its derived protein sequence overlap with the downstream, partial VP ORF, indicating that the intervening assembly gap region may correspond to the missing portion of the VP gene.

Splicing of a putative intron sequence may position three residues of the short, 23-aa-long upstream adjacent ORF in-frame with the VP ORF, consistent with the typical VP1u transcription pattern of amdoparvoviruses (Qiu et al. 2006). The putative VP ORF encoded by EllLut.1 has a gap relative to the AMDV VP that spans most of the 5′ region of the gene. Frameshifting mutations are present in the NS genes of both elements, and the VP pseudogenes of EllLut.1 (Fig. 1).

Amdoparvovirus genomes encode a short middle ORF (M-ORF) of unknown function between the two major ORFs (NS and VP) (Bloom et al. 1988; Gottschalck et al. 1994; Li et al. 2011). As shown in Fig. 1, a region of potentially protein-coding sequence that corresponds to the M-ORF of AMDV is present in the EllLut.1 element. A methionine (M) residue that might represent the start codon of an M-ORF gene product could not be identified. However, this is also the case for several exogenous amdoparvovirus isolates (Gottschalck et al. 1994; Li et al. 2011).

The EllLut.2 element comprises the NS gene alone (Fig. 1). This element is integrated into a locus immediately adjacent to the sequences encoding the MAF BZIP transcription factor G (MAFG) gene, which in the mouse genome is located in the 11qE2 region of chromosome 11. The structure of the EllLut.2 element indicates that it was derived from an NS-encoding mRNA that was reverse transcribed and integrated into the nuclear genome of an ancestral germline cell. The otherwise intact NS gene lacks a methionine start codon and 5′ UTR. However, immediately upstream of the three stop codons disrupting the MAFG gene, a conventional ATG start codon was identified that could provide translation initiation to express a MAFG-NS fusion product (Fig. 1). The identification of a potential promoter sequence downstream of the MAFG gene supports the existence of such a fusion protein, as does the strong Kozak translational context of the above mentioned start codon.

We identified empty integration sites in the *Ellobiustalpinus* genome at the loci where the EllLut.1 and EllLut.2 elements are integrated in *E. lutescens*. This indicates that both elements were integrated into the *E. lutescens* germline after these two species diverged ∼10 million years ago (MYA) (Fabre et al. 2012; Pisano et al. 2015). The genomes of two *E.lutescens* individuals have been generated (genomic DNA was obtained from the livers of both a male and a female individual). Both EPV sequences were present in both individuals. However, the EllLut.2 element in the female animal had a 13–14 bp deletion relative to the one in the male.

### 3.3 An EPV in the pit viper genome with amdoparvoviral and protoparvoviral characteristics

We identified an EPV in the genome of the spotted pit viper (*Protobothrops mucrosquamatus*), which we labeled EPV-AP-ProMuc.1 (ProMuc.1). This element, which encoded a nearly complete parvovirus genome, was ∼4.5 kb in length and was integrated in reverse orientation (i.e. preserving the presumed original negative orientation of the virus genome). The putative genome structure comprised two major ORFs and a minor ORF as well as a clearly-identifiable and potentially functional downstream promoter (Fig. 1). Furthermore, two polyadenylation signals could be identified, as well as partial palindromic repeats resembling the amdoparvoviral hairpin structures in the expected positions upstream and downstream of the two ORFs (Fig. 1 and Supplementary Fig. S1).

The first major ORF exhibited a relatively high degree of aa identity to the AMDV NS protein (35% with no deletions). The putative peptide gene product clustered as a outgroup to a clade containing the mole vole EPVs and exogenous amdoparvoviruses (Fig. 2a), but only with weak bootstrap support. The second major ORF, which was disrupted by several nonsense mutations (two stop codons and two frameshifts), was homologous to VP (36% aa identity with skunk amdoparvovirus VP). This ORF did not possess a conventional Met start codon to express VP1; however, the three aa-long exon leader of a short upstream ORF could potentially provide this, as in other amdoparvoviruses (Qiu et al. 2006). A putative middle (M) protein ORF was identified between the putative NS and VP genes.

**Figure 3.**
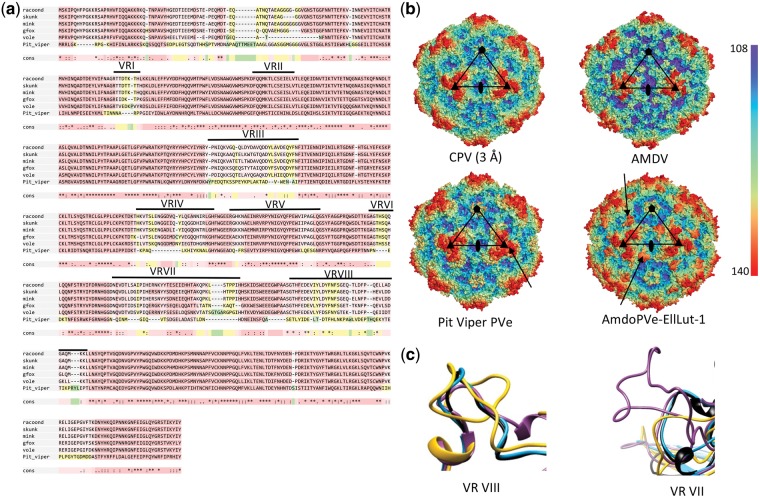
(a) Alignment of the EPV VP protein sequences with those of exogenous amdoparvoviruses. VRs are indicated by horizontal lines. (b) Results of homology modeling; the capsid structure of CPV served as a reference structure for all the three further models. The bar shows the distance from the capsid center in Ångströms and the structures are colored accordingly. The pentagon marks the fivefold, the triangles the threefold, and the twofold is indicated by an ellipse. The arrows mark the VRIII region of ProMuc.1 and the VRVII of the EllLut-1 capsids, which contain the only insertions compared to amdoparvovirus VR regions. (c) Ribbon diagrams of the VRVIII (left) and VRVIII (right) loops of CPV (blue), AMDV (black), EllLut-1 (pink), and ProMuc.1 (yellow). racoond, racoondog amdoparvovirus; gfox, grey fox amdoparvovirus; skunk, skunk amdoparvovirus; mink, AMDV; vole, Transcaucasian mole vole EPV EilLut.1; Pit_viper, pit viper EPV ProMuc.1.

The polyglycine (poly-G) region in parvovirus VP proteins is suspected to be responsible for externalizing the VP1u (so that the enzymatic functions of the PLA2 domain can be carried out) as well as exposing the nuclear localization signal (NLS) of the VP1u and the VP2 (Chapman and Rossmann 1993; Vihinen-Ranta et al. 2002). All exogenous amdoparvovirus VP peptide sequences contain a poly-G, despite lacking a PLA-2 domain. A poly-G region was also present in the predicted VP sequence of ProMuc.1, whereas it was absent from EllLut.1 VP (Supplementary Fig. S2). Interestingly, however, the VP1u sequences of both EPVs disclosed a putative NLS. Notably, the ProMuc.1 VP sequence contained numerous indels relative to those of amdoparvoviruses. Notably, however, indels were almost exclusively confined to the VR loops (Fig. 3). The only insertion, six-aa-long, was present in VR VIII. Interestingly, dependoparvovirus-derived EPVs previously identified in marsupial genomes have also been reported to harbor extended VRVII loops (Smith et al. 2016).

ProMuc.1 occurs on a contig that has not been mapped to a specific chromosome. Nevertheless, the preintegration locus could be identified in WGS data of two other reptilian species: the Burmese python (*Python bivittatus*), and a colubrid, the common garter snake (*Tamnophis sirtalis*) (data not shown). The absence of a ProMuc.1 insertion in these taxa establishes that it was incorporated into the germline of the pit viper subsequent to its divergence from these species, which is estimated to have occurred 34–54 MYA (Head et al. 2005).

### 3.4 EPVs in mammalian genomes with amdoparvoviral and protoparvoviral characteristics

Genome screening *in silico* identified three additional matches to amdoparvoviruses in mammal genomes (Table 1). We examined these Pve and found that all three of were highly fragmented by stop codons, frameshifts, and transposable elements. Nevertheless, all three encoded near complete VP peptides, all of which exhibited a well-preserved calcium-binding loop in their N-terminal PLA2 domains (Fig. 1). However, the catalytic core was barely recognizable in the Cape hyrax element (ProCap.1) and completely absent in the aarvark element (OryAfe.1) (Fig. 1b and Supplementary Fig. S2). ProCap.1 appeared to lack an NLS sequence, and a poly-G stretch was absent from all three elements, as in the VP encoded by the amdoparvovirus-derived EPV EllLut.1 (Supplementary Fig. S2). Apart from the disintegrated catalytic domain of the PLA2, the Tasmanian devil element (SarHar.1) displayed the most well-preserved VP1u sequence.

With the exception of OryAfe.1, which contained a highly disrupted NS homolog spanning 343 aa residues, only a minimal trace of the nonstructural genes could be detected (Fig. 1). In phylogenies based on NS (Fig. 2a), OryAfe.1 grouped together with the Mpulungu bufavirus of shrews (Sasaki et al. 2015) as a robustly supported sister group to rodent, ungulate and carnivore protoparvoviruses. In phylogenies based on VP (Fig. 2b), all three mammal EPVs formed a robustly supported clade in a position intermediate between the amdoparvoviruses and protoparvoviruses. The viper element ProMuc.1 grouped basal to this clade, but with weak support.

### 3.5 Structural characterization of EPV capsid proteins via homology modeling

We investigated the capsid (VP) sequences of the more complete and intact EPVs using homology modeling. Using this approach, the capsids encoded by pit viper and mole vole EPVs proved to be structurally most similar to the canine parvovirus (CPV) capsid (PDB ID: 2CAS) according to fold recognition, hence this structure was used as a template. As there are currently no publicly available structural data for amdoparvorviruses, we constructed the model of the AMDV capsid as well, based on the CPV template.

The predicted structures of the capsid proteins encoded by the ProMuc.1 and EllLut.1 displayed a rather protoparvovirus-like appearance, unlike the AMDV capsid model (Fig. 3). In the case of ProMuc.1, threefold protrusions were thicker and bulkier than either on CPV or AMDV, while the EllLut.1 capsid model displayed spike-like protrusions rather than the slope-like depressions characteristic of the parvovirus two-/fivefold wall. These differences could be ascribed to insertions in VRs, namely VRVIII of the ProMuc.1 and VRVII of EllLut.1. Both capsids appeared to contain the canonical β-strand A (βA), an eight-stranded β-barrel core making up the jelly roll fold (βBIDG-CHEF), and an α-helix (αA) (Fig. 3b and c).

## 4. Discussion

### 4.1 The genomic fossil record of amdoparvoviruses

The assorted EPV sequences in animal genomes are a unique and useful source of retrospective information about parvovirus evolution, in some ways equivalent to a parvovirus ‘fossil record’ (Katzourakis and Gifford 2010). However, while there are eight parvovirus genera currently recognized to infect vertebrates (Cotmore et al. 2014), almost all the EPVs that have been identified in vertebrate genomes are derived from just two genera: *Dependoparvovirus* and *Protoparvovirus*. EPVs derived from dependoparvoviruses have been identified in several orders of birds and mammals (Belyi et al. 2010; Katzourakis and Gifford 2010; Cui et al. 2014), while EPVs derived from protoparvoviruses have been reported in rodent genomes (Kapoor et al. 2010). Large numbers of ‘protoparvovirus-like’ EPVs are present in the genomes of mammals, including rodents and marsupials (Katzourakis and Gifford 2010; Arriagada and Gifford 2014), but it is less clear whether these derive from *bona fide* protoparvoviruses or a distinct parvovirus lineage (e.g. an extinct genus). Prior to this study, only a single, highly fragmented EPVs (ProCap.1) had been reported as showing homology to amdoparvoviruses (Katzourakis and Gifford 2010).

We report the first EPVs that are unambiguously derived from amdoparvoviruses. These two elements, which were identified in the genome of the Transcausian mole vole, were found to group within the diversity of amdoparvovirus isolates in molecular phylogenies (Fig. 2). They also exhibit characteristic features that support their grouping within the genus *Amdoparvovirus*, including the presence of a putative M-ORF, and the absence of the PLA2 domain from the predicted VP protein sequence (Fig. 1 and Supplementary Fig. S2).

We did not identify any other EPVs that grouped convincingly within the *Amdoparvovirus* genus. However, we identified several that displayed a mixture of amdoparvoviral and protoparvoviral features. EPVs in this ‘amdo-proto’ (AP) group—which may not be monophyletic (see later)—grouped in an intermediate position in phylogenetic trees. Furthermore, while these elements appear to be marginally more closely related to protoparvoviruses than to amdoparvoviruses (Fig. 2), certain aspects of their genome organization suggested an evolutionary connection to amdoparvoviruses. For example, in the pit viper element, these include: (1) the presence of a single promoter and two polyadenylation signals; (2) the attributes of the intron in the VP1u; and (3) the apparent absence of a PLA2 domain (Fig. 1).

We were able to infer maximum age bounds of 54 million years for the pit viper EPV and 10 million years for the mole vole EPVs, based on the identification of empty integration sites in related species. However, none of the EPVs reported here were identified as orthologous copies in two or more related species. Consequently, we are unable to draw firm conclusions with respect to their minimum ages. The mutational degradation observed in the more fragmented elements suggests they are likely to have similarly ancient origins to other EPVs (i.e. extending back millions of years). In the case of the mole vole, two EllLut.2 alleles were present (one containing a deletion relative to the other), indicating that these elements have likely been present in the species gene pool for multiple generations.

The confirmed host range of amdoparvoviruses is restricted to carnivores, but this likely reflects limited sampling—metagenomic studies of parvovirus diversity are providing strong hints that most if not all genera in the subfamily *Parvovirinae* are likely to have representatives that infect all most if not all extant mammalian orders (de Souza et al. 2018). Nonetheless, the identification of amdoparvovirus-derived EPVs in the genome of the Transcaucasian mole vole advances our current knowledge by uneqivocally demonstrating that amdoparvoviruses have infected rodents in the past. The pit viper element reported here (ProMuc.1) is the first EPV to be identified in a reptile genome.

### 4.2 Evolution of amdoparvovirus and protoparvovirus capsid proteins

Comparison of predicted VP protein structures to those of exogenous amdoparvoviruses revealed that most differences are limited to regions of conspicuous functional significance, including the PLA2 domain, VP1u, and the VR loops that are exposed on the virion surface and are thought to be involved in mediating many host-virus interactions, such as immunogenicity and receptor attachment (Huang et al. 2014). The fact that variation is overwhelmingly confined to these regions suggests it largely reflects diversity accumulated through selection on ancestral viruses, rather than mutations acquired postintegration. The strikingly high level of deletions seen in the VR regions of the ProMuc.1 VP protein is consistent with this, since we might expect that the reptilian anti-viral response, in which the adaptive immune system plays a relatively small role, would exert selective pressures that were somewhat different to those encountered by parvoviruses infecting mammals (Zimmerman et al. 2010). A similar idea has previously been proposed to explain the characteristically smooth surface features, i.e. shorter or absent VRs, of invertebrate-infecting densoviruses (Simpson et al. 1998).

Uniquely, amdoparvoviruses lack a PLA2 domain in their VP1u region (Cotmore, 2014, p. 22). Most parvoviruses require this highly conserved motif for escape from the endosomal compartments after entering the host cell (Zadori et al. 2001), and consequently amdoparvoviral trafficking is not fully understood. EPVs in the AP lineage are presumably derived from an uncharacterized parvovirus lineage possessing amdoparvovirus and protoparvovirus-like features. However, the lack of phylogenetic resolution between the three main clades, and the fragmentary genome structures of most elements in the AP lineage, limits what we can infer about them. They might derive from viruses representing transitional forms along a pathway from protoparvovirus-like complete PLA2 domains to an amdoparvovirus-like PLA2-absent state. However, they could also represent an entirely distinct lineage that diverged from a common ancestor shared with contemporary amdo- and protoparvoviruses. In fact, since the pit viper element only groups weakly with other AP elements in phylogenetic trees, the AP clade shown in Fig. 2 may not in fact be truly monophyletic, and might instead represent entirely distinct mammalian and reptilian lineages.

Interestingly, an intact calcium binding loop was found in the predicted VP1u protein sequences of all EPVs in the AP lineage that encoded this region, even though most of these sequences also have a degraded PLA2 domain (Fig. 2 and Supplementary Fig. S2). This observation raises the possibility that this loop might have functions in the viral life cycle that are unrelated to its role in phospholipase-mediated escape from the endosomal compartments.

We used homology modeling to infer the structures of the capsid (VP) proteins encoded by EPVs. This analysis revealed that both EilLut.1 and ProMuc.1 capsids had a protoparvovirus-like appearance, rather than being similar to the AMDV capsid (Fig. 3). These findings are intriguing when considered in the light of the phylogenetic relationships depicted in Fig. 2. Results based on homology modeling should of course be interpreted cautiously, but these observations could reflect that the ancestral viruses that gave rise to these two elements were more similar to protoparvoviruses than amdoparvoviruses in certain aspects of their biology related to their capsid proteins (e.g. tropism or receptor specificity). With regard to this, the prominent role of Fc-receptor-mediated antibody-dependent enhancement (ADE) in AMDV infection should be considered (von Kietzell et al. 2014). Residues 428–446 in VP have been shown to play an important role in mediating Fc-receptor attachment during ADE, and interestingly this region overlaps with VR VII, which is highly divergent in both ProMuc.1 and EllLut.1 (Fig. 3). It is not known whether ADE plays an important role in infections with amdoparvoviruses other than AMDV, or whether the variability observed in this region is relevant to this process, but it is nonetheless intriguing to consider that the distinctive appearance of the AMDV capsid might be related to its use of ADE as an entry mechanism.

### 4.3 Intact, potentially expressible NS genes encoded by EPVs reported here

Two of the EPVs described here (EilLut.2 and ProMuc.1) encode intact, expressible NS genes, adding to a growing number of EPVs that exhibit this characteristic (Katzourakis and Gifford 2010; Liu et al. 2011; Arriagada and Gifford 2014). Recent studies have shown that independently acquired EPVs in rodents and afrotherian genomes exhibit similar patterns of tissue-specific expression in the liver (Arriagada and Gifford 2014; Kobayashi et al. 2018), suggesting that EPVs may have been co-opted or exapted by mammalian genomes on more than one occasion. Intriguingly, *in silico* predictions indicated that the intact EilLut.2 replicase identified here could be expressed as a fusion protein with a partial MAFG gene product (Fig. 1a).

## Supplementary Material

Supplementary Figure 1

Supplementary Figure 2

Supplementary Table 1
