# Supplementary material for: Endogenous amdoparvovirus-related elements reveal insights into the biology and evolution of vertebrate parvoviruses
Source: Virus Evol. 2018 Nov 12;4(2):vey026. doi: 10.1093/ve/vey026 (PMC6232428; doi:10.1093/ve/vey026)
Supplement: Supplementary Figure 1 [file vey026_supp_fig_s1.docx]

**
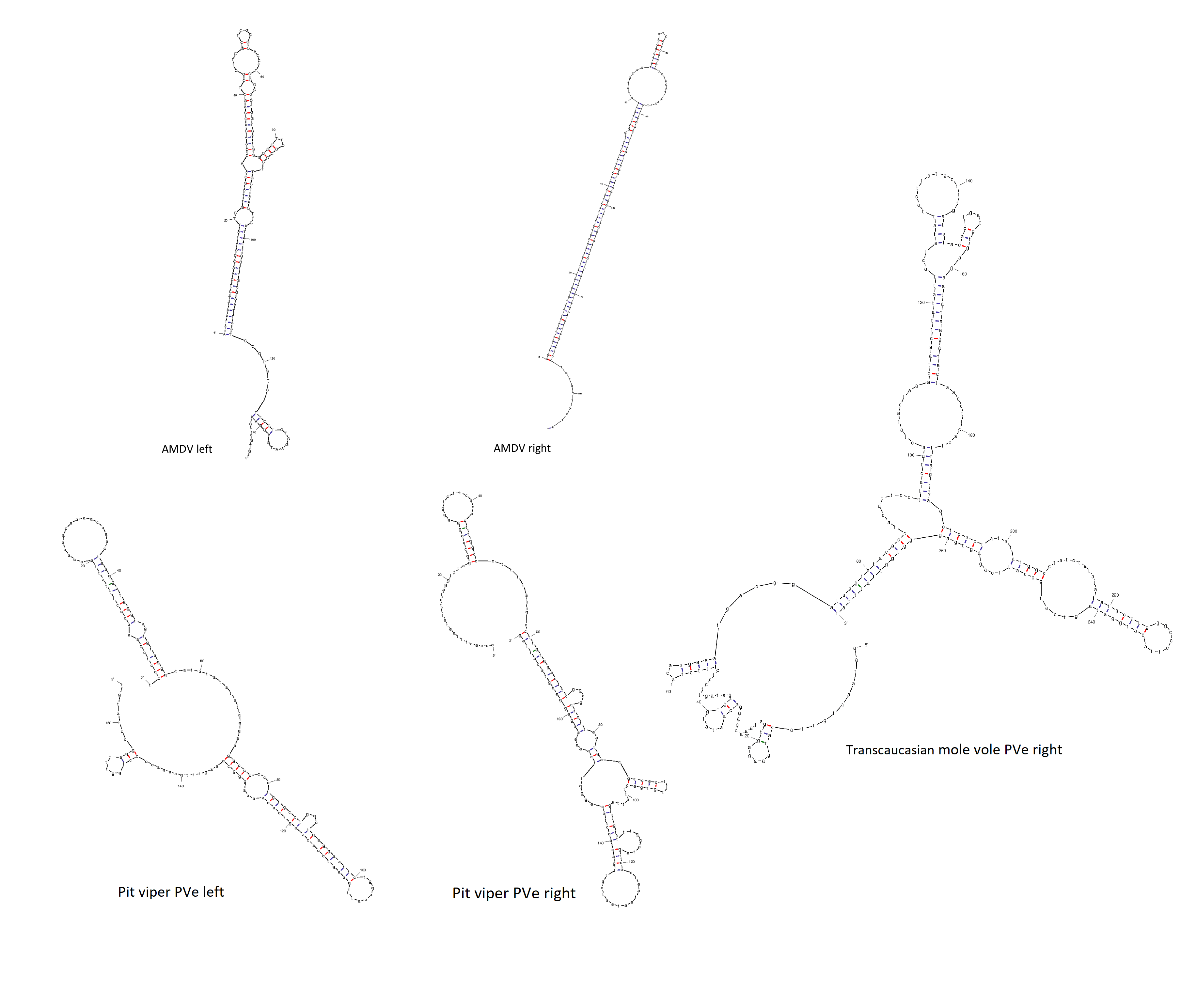
**

**Figure S1. Hairpin structures identified in PVe.**

Hairpin-like structures identified directly downstream of the promoter (5’ hairpin) and upstream of the last polyadenylation signal (3’ hairpin) in the pit viper endogenous viral element ProMuc.1. A 3’ hairpin structure detected in the Transcaucasian mole vole element EilLut.1 is also shown. The predicted Aleutian mink disease virus (AMDV) termini secondary structures are provided for comparison.
