# Supplementary material for: Endogenous amdoparvovirus-related elements reveal insights into the biology and evolution of vertebrate parvoviruses
Source: Virus Evol. 2018 Nov 12;4(2):vey026. doi: 10.1093/ve/vey026 (PMC6232428; doi:10.1093/ve/vey026)
Supplement: Supplementary Figure 2 [file vey026_supp_fig_s2.docx]

Mpulungu bufavirus 1 M-PLLKGF------------------------KD**GLTLPGYNYLGPGNSDFTKKPTNASDAAARKHDLAYGSYLKKGHNPYINFNNADKTFIK**DT----------   70 
h1                  1 MAPPAKRA------------------------KR**GWVPPGYKYLGPGNSLDQGEPTNPSDAAAKEHDEAYDQYIKSGKNPYLYFSAADQRFID**QTK---------   72 
canine parvovirus  1 MAPPAKRARRGKGVLVKWGEGKDLITXLSMCFFI**GLVPPGYKYLGPGNSLDQGEPTNPSDAAAKEHDEAYAAYLRSGKNPYLYFSPADQRFID**QTK---------   96 
porcine parvovirus 1 MAPPAKRA-------------------------R**GLTLPGYKYLGPGNSLDQGEPTNPSDAAAKEHDEAYDKYIKSGKNPYFYFSAADEKFIK**ETE---------   71 
PVe-SarHar-1    1 --------------------------------MQ**NYTLPGYNYLRPGNTGHEQKLTSQCDKAAKLHVQAYXQ-IDQGHNPYLYFNKTDKNFIE**ATKETSXRLPPE   72 
PVe-OryAfe-1    1 F--------------------------------I**GYATSGYNYLGPGNTLQSQKKKKKKPNPCD-QLQKH-KMRKKGKNLYIFHNVTDDKLTQ**ATE---------   62 
PVe-ProCap-1    1 MIIYSYNIL---------------------IYCK**LYVLPGYYYLVPGNILEGKKTTKPPDEAVKKHDQGYILLTEQDLNPYLLHNESDHKLIK**ATN---------   75 
PVe-EllLut-1    1 M--------------------------------------------------------------------------------------------------------    1 
PVe-ProMuc-1    1 M--------------------------------------------------------------------------------------------------------    1 
racoondog amdoPV    1 M--------------------------------------------------------------------------------------------------------    1 
skunk amdoPV        1 M--------------------------------------------------------------------------------------------------------    1 
AMDV        1 M--------------------------------------------------------------------------------------------------------    1 
Grey fox amdoPV     1 M--------------------------------------------------------------------------------------------------------    1 

cons                1                                                                                                            105 


Mpulungu bufavirus 71 ----NQAK----------DWGGWLGNKFFKIKEHIAPKLPEEPP-TKKPRPSTSKDPKYSWRHLKEGT----KKPKPFWLFVNKARQKKS---MDGGTST---DT  150 
h1                 73 -----DA----------KDWGGKVGHYFFRTKRAFAPKLATD-S-----EPGTSGV-S-----R-AGK----RTRPPAYIFINQARAKKK---LTSSAAQQSSQT  142 
canine parvovirus  97 -----DA----------KDWGGKIGHYFFRAKKAIAPVLTDTPD-----HPSTSRP-T-----K-PTK----RSKPPPHIFINLAKKKKA---GAGQVKRDNLAP  167 
porcine parvovirus 72 -----HA----------KDYGGKIGHYFFRAKRAFAPKLSETDSPTTSQQPEVRRSPR-----KHPGSKPPGKRPAPRHIFINLAKKKAK---GTSNTNSNSMSE  153 
PVe-SarHar-1    73 NLYTNQLQWLNTLXRFQQQATGEVALKVFKVKELLLPKLTPPI---KKAKT------QFHLTHF-GKT----NKQVPRHVFIKQARKIXSRKLSQSNKEN---PS  160 
PVe-OryAfe-1    63 -----DW-------------GGKVAHCVFKTKRTLVPKTPTSLKIKXINKXYKQNYFYPRCRSY-RGI----KQVAPKHIFVNLAKRRVQ---QVNXQNTKQLLL  141 
PVe-ProCap-1    76 -----NA--------------------------------------------------------------------------------------------------   77 
PVe-EllLut-1    2 ------------------------------------PRVPQ---------------------H-YPGK----KRSAPRFVFVQQAKKKKQ---SNPAVSH-EELT   40 
PVe-ProMuc-1    2 ------------------------------------RRLGK---------------------R--PG----------KHIFINLARKKSKQ--SSQQTSE-DPLE   34 
racoondog amdoPV    2 ------------------------------------SKIPQ---------------------H-FPGK----KRSAPRHVFIQQAKKRKQ---TNPAVFH-GEDT   40 
skunk amdoPV        2 ------------------------------------SKIPQ---------------------H-YPGK----KRSAPRHVFIQQAKKKKQ---TNPAVYH-GPDT   40 
AMDV        2 ------------------------------------SKIPQ---------------------H-YPGK----KRSAPRHVFIQQAKKKKQ---TNPAVYH-GEDT   40 
Grey fox amdoPV     2 ------------------------------------SKIPQ---------------------H-YPGK----KRSAPRHVFIQQAKKKKQH--SNPAVSH-QEDT   41 

cons              106                                                                                                            210 


Mpulungu bufavirus151 QDE--------PNDAAATASNTGT-------TGGGGGGGSGVGHS-TGNFDNRTDFRYE-NGEVTIICRATRLVHVKMSDSEEYRIFQTNNGDQFPP-STTRNEA  237 
h1                143 MSD-GTS-QPDSGNAVHSAARVERAADGPGGSGGGGSGGGGVGVS-TGSYDNQTHYRFLGDGWVEITALATRLVHLNMPKSENYCRIRVHNTTDTSV-------K  237 
canine parvovirus 168 MSD-GAV-QPDGGQPA-VRNERAT-GSGNGSGGGGGGGSGGVGIS-TGTFNNQTEFKFLENGWVEITANSSRLVHLNMPESENYRRVVVNNMDKTAV-------N  260 
porcine parvovirus154 NVE-QHN-PINAGTEL-SA--TGN-ESGGGGGGGGGRGAGGVGVS-TGTFNNQTEFQYLGEGLVRITAHASRLIHLNMPEHETYKRIHVLNSE-SGV-------A  243 
PVe-SarHar-1    161 MTE--------QMQQLPLRENSQE-------QAGLGNGGRKVGSRYLNRLHQCSPXNYN-KG------HASRYINQSMPKNERYTYYKKVETQFTGT-GAT----  238 
PVe-OryAfe-1    142 NKQ-WRP-KTLQMMQ--VVSKLLX--DL---R-ALVGEEREVSLN-IGDFVNTTEWKYN-NGHVYITCHTSRLIHLNMPENEEYTLQQITTTF-T-T-------I  225 
PVe-ProCap-1    78 -----------------------K--DG---E-I-------KKKQ-TLFFTKTIKWNFD-NGCVYVTCHASRGVHLNMPGSEGYTSPKMPKTL-S-A-------T  135 
PVe-EllLut-1    41 VEEME---EPEQMDTG---------------------------EQ-ATNFNNTTEFKVT-NNEVTITCHATRVVHINQADTDEYLIFNAGRTVEDKPVYRDSLDL  113 
PVe-ProMuc-1    35 GTSQDTHHSPTVMDNAPAQTTMEETAAGLG-GASGGMGGGGVGLS-TGGLRSTIEWKHLGGGEILITCHSSRLIHLNMPESEEYKMLTINNNARPPG----IEYI  133 
racoondog amdoPV   41 IEEMDST-EPEQMDTEQATNQTAEAG----------GGGGGVGNS-TGGFNNTTEFKVI-NNEVYITCHATRMVHINQADTDEYVIFNAGRTTDTKT-HLKKLNL  131 
skunk amdoPV       41 IEEMDPA-EPEQMDTEQATNQTAEAG-------------GGVGNS-TGSFNNTTEFKVI-NGEVYITCHSTRMVHINQADTDEYLVFNAGRTTDTTT-THDKLDL  128 
AMDV       41 IEEMDST-EAEQMDTEQATNQTAEAGGGGGGGGGGGGGGGGVGNS-TGGFNNTTEFKVI-NNEVYITCHATRMVHINQADTDEYLIFNAGRTTDTKT-HQQKLNL  141 
Grey fox amdoPV    42 IEEMDSN-ETEQMDISEQ-QQVAEHG-------------GGVGKS-TGGFNNTTEFKVT-NNEVIITCNATRVVHINQASTDEYLIFNAGREIDKTP--KGSLNL  127 

cons              211                                                    .    .    .       ::* :: . .  : *                       315

**Figure S2. Multiple sequence alignment of minor capsid protein (VP1) sequences.**

The alignment spans the N-terminal region of the VP1 and includes representative protoparvoviruses (yellow), amdoparvoviruses (cyan), and endogenous parvoviral elements (PVe). The VP1 unique (VP1u) region and the phospholipase A2 (PLA2) domain (if present) are indicated by bold text. The calcium-binding site is marked with blue arrows, while black arrows highlight the catalytic core. A glycine-rich stretch (framed) is present at the beginning of the major capsid protein VP2-overlapping region. This region is suspected to mediate conformational changes during endosomal escape. Similarly to contemporary amdoparvoviruses, the pit viper and vole PVe completely lack the PLA2 domain, whereas the catalytic core is absent in case of the OryAfe.1. Remnants of the G-rich region are recognizable in case of SarHar.1, but entirely absent in the other mammalian PVe. The putative nuclear localization signal (NLS) is underlined. An NLS is present in all PVe except ProCap.1. Abbreviations: PV=parvovirus; H1=H-1 parvovirus. For details of PVe see **Table 1**. For details of the amdoparvoviruses and protoparvoviruses examined here see **Table S1**.
