# Supplementary material for: Endogenous amdoparvovirus-related elements reveal insights into the biology and evolution of vertebrate parvoviruses
Source: Virus Evol. 2018 Nov 12;4(2):vey026. doi: 10.1093/ve/vey026 (PMC6232428; doi:10.1093/ve/vey026)
Supplement: Supplementary Table 1 [file vey026_supp_table_s1.docx]

**Table S1. Parvovirus reference sequences**

| **Virus** | **Genus** | **Accession number** |
| --- | --- | --- |
|  |  |  |
| Skunk amdoparvovirus strain SK-23 | Amdoparvovirus | NC_034445.1 |
| Gray fox amdovirus | Amdoparvovirus | JN202450.1 |
| Raccoon dog amdovirus | Amdoparvovirus | KJ396350.1 |
| Aleutian mink disease virus | Amdoparvovirus | NC_001662.1 |
| Canine parvovirus | Protoparvovirus | NC_001539.1 |
| Porcine parvovirus | Protoparvovirus | D00623.1 |
| Minute virus of mice | Protoparvovirus | NC_001510 |
| Bat parvovirus | Protoparvovirus | KJ641666 |
| H-1 parvovirus | Protoparvovirus | JX505432 |
| Kilham rat virus | Protoparvovirus | AF321230 |
| Hamster parvovirus | Protoparvovirus | U34255 |
|  |  |  |
